# Polypharmacy and health related quality of life among older adults on antiretroviral therapy in a tertiary hospital in Tanzania: a hospital-based cross-sectional study

**DOI:** 10.1186/s12879-023-08150-x

**Published:** 2023-03-27

**Authors:** Antimon Tibursi Massawe, Grace Ambrose Shayo, Sabina Ferdinand Mugusi

**Affiliations:** 1 Department of Internal Medicine, Muhimbili University of Health and Allied Sciences, P.O.BOX 65001, Dar Es Salaam, Tanzania; 2 Department of Clinical Pharmacology, Muhimbili University of Health and Allied Sciences, P.O.BOX 65001, Dar Es Salaam, Tanzania

**Keywords:** HIV, Polypharmacy, Older adults, Quality of life, Co-morbidities

## Abstract

**Background:**

Human immunodeficiency virus (HIV) chronicity in the midst of old age multiplies the risk for chronic non communicable diseases. The old are predisposed to drug-drug interactions, overlapping toxicities and impairment of the quality of life (QoL) due to age-related physiological changes. We investigated polypharmacy, QoL and associated factors among older HIV-infected adults at Muhimbili National hospitals in Dar es Salaam Tanzania.

**Methods:**

A hospital-based cross sectional study enrolled adults aged 50 years or older who were on antiretroviral therapy (ART) for ≥ 6 months. Participants’ Information including the number and type of medications used in the previous one week were recorded. Polypharmacy was defined as concurrent use of five or more non-HIV medications. A World Health Organization QoL questionnaire for people living with HIV on ART (WHOQoL HIV BREF) was used to assess QoL. A score of ≤ 50 meant poor QoLwhile > 50 meant good QoL. Polypharmacy and QoL are presented as proportions and compared using Chi-square test. Association between various factors and polypharmacy or QoL was assessed using modified Poisson regression. A *p*-value of < 0.05 was considered significant.

**Results:**

A total of 285 patients were enrolled. The mean (SD) age was 57(± 6.88) years. Females were the majority (62.5%), and 42.5% were married. Polypharmacy was seen in 52 (18.2%) of participants. Presence of co-morbidities was independently associated with polypharmacy (*p* < 0.001). The mean(SD) score QoL for the study participants was 67.37 ± 11.Poor QoL was seen in 40 (14%) participants.All domains’ mean score were above 50, however social domain had a relatively lowmean scoreof 68 (± 10.10). Having no formal or primary education was independently associated with poor QoL (*p* = 0.021).

**Conclusion:**

The prevalence of polypharmacy was modestly high and was linked to the presence of co-morbidities. No formal and/or primary education was associated with poor QoL, where by social domain of QoL was the most affected.

## Background

Human immunodeficiency virus infection (HIV) is still a global burden despite intensive efforts to stop the chain of transmission. In the year 2021, Globally 38.4 million people were living with HIV, 28.7 million people were accessing antiretroviral therapy, 1.5 million people became newly infected and 650 000 people died from AIDS-related illnesses [1].

In the year 2019, Tanzania had an estimated 1.7 million people living with HIV (PLWHIV), making a prevalence of 4.8% among people aged 15–49 years. As it has been over the past years, women were more affected (6.0%) than men (3.6%). The country reported 27,000 AIDS-related deaths despite having ART coverage of about 75% among all adults living with HIV [2]. The data for the year 2020 was more or less comparable to 2019 data, with a report of about 58,000 new infections, 50% of these were among youths aged 15–29 years [3]. A cross-sectional study in northern Tanzania by Swai et al. (2015) reported a prevalence of HIV to be 1.7% among people aged 50 years or older in year [4].

Knowing that Anti-Retroviral Therapy (ART) has transformed HIV disease from a deadly disease to a chronic manageable condition [5, 6], it is irrefutable that ART has increased life expectancy among PLWHIV [7]. PLWHIV are now living longer to experience age-related co-morbidities like diabetes, arthritis, and cardiovascular diseases thus expose them to multiple drugs use [8]. HIV by itself and its treatment are associated with a number of non-communicable diseases such as atherosclerosis, arterial stiffening, hypertension, dyslipidemia and thus may result into complications associated with cardiovascular diseases [9–11]. Cardiovascular complications associated with HIV and its treatment includes acute coronary syndrome, stroke, and vascular diseases. Other co-morbidities are intestinal and renal diseases and many others. [12]. Non-nucleoside reverse transcriptase inhibitors (NNRTIs) are known to have side effects like ischaemic heart disease, peripheral neuropathy and pancreatitis; Protease inhibitors (PIs) do cause hyperlipidemia, diabetes and hepatitis. [13]. Tenofovir disoproxil fumarate may cause chronic kidney disease which may be worse enough to necessitate renal replacement therapy [14, 15].

The World Health Organization (WHO) defines polypharmacy in a general population as the use of five or more drugs [16]. Among HIV infected individuals who are on ART, the WHO defines polypharmacy as the concurrent use of five or more non-HIV medications [17, 18].

Polypharmacy is among the burdens faced by HIV infected individuals on ART [17]. It is observed more in older adults, including those on ART [19, 20], possibly because older adults receiving ART are faced with both age-related (non-HIV) and HIV-related co-morbidities [8, 21].

Polypharmacy increases chances of drug-drug interactions and hence adverse drug effects to both the young and the old, however old PLWHIV are more at risk for side effects of polypharmacy hence vulnerable to geriatric syndromes [19, 22–25]. Old people have altered pharmacokinetics due to reduced residual function of different body organs [26, 27], The main mechanisms involved in drug-drug interactions among PLWHIV on ART are those which affect the pharmacokinetics of drugs at different stages including absorption, protein binding, hepatic metabolism and drug transporter molecules [26]. Age-related co-morbid conditions are the leading factors contributing to polypharmacy [27, 28]. Regardless of the presence of age-related co-morbidities, polypharmacy has been found to be associated with increased risk of falls, fractures, frailty, delirium, pills burden and hence poor medication use [29–31] and poor quality of life (QoL) outcomes. Medication side effects have been reported to be the main cause of care dissatisfaction [32], an entity largely linked to polypharmacy. Furthermore polypharmacy is associated with poor patient satisfaction, reduced QoL and poor clinical outcomes including poor viral load suppression and immunological improvement [31].

Polypharmacy among the elderly may be minimized in a number of ways which include thorough medications history, review of indication for the current medications, good communication with healthcare providers, review of drug-drug interaction and determination of therapeutic ratio [27].

In Tanzania, there is scarcity of data on the magnitude of polypharmacy, associated factors and its effect on health-related QoL among HIV-infected older adults on ART. This study was done to inform on the burden and risk factors for polypharmacy and its impact on the QoL among older adults on ART.

## Methods

### Study design and study area

This was a hospital-based cross sectional study conducted at Muhimbili National Hospital situated in Dar es Salaam city, Tanzania from July 2021 to December 2021. It is the largest tertiary hospital in the country with two campuses Upanga and Mloganzila. It also serves as a teaching hospital for the Muhimbili University of Health and allied sciences. CTC clinic at MNH Upanga provides care for both children and adults with more than 3000 enrolled PLWHIV. In Mloganzila campus, the CTC had enrolled about 300 clients. The two clinics operate for 5 days in a week i.e. Mondays to Fridays excluding public holidays.

### Study population

All HIV-infected out-patients aged 50 years or older, on ART for 6 months or more attending care and treatment clinics at Muhimbili National Hospital, Upanga and Mloganzila campuses. We excluded participants who did not provide consent and the very sick who needed emergency care as it wasn’t possible to interview them.

### Study procedures

Participants were informed about what the study was all about and asked for their consent to participate. Consenting participants were enrolled consecutively until the required sample size was attained. Data was collected using a face-to-face interviewer-administered structured questionnaire inquiring about socio-demographic and clinical data including co-morbidities and number of medications (including supplements) they were using concomitantly with their ART in the past one week. Participants were also required to provide their active phone contacts at the time of interviews. Those who were not able to recall their medications at the time of interview were contacted later via their phone contact to send names (for those without smart phones) or pictures of the medication leaflets and/or packages to be able to ascertain the number and type of medications they were using. Traditional medications were not counted since their chemical composition and effect/drug-drug interactions are un-predictable.

We recorded information on co-morbid conditions and the number of other regular clinics participants were attending. A short version of the World Health Organization (WHO) QoL questionnaire for HIV-infected patients on ART (WHO QoL-HIV BREF) was used to assess participants’ QoL. The WHOQoL-HIV BREF has six domain scores made up of 29 items plus two items inquiring about individuals’ general QoL(Overall QoL and health perception) which then makes a total of 31 items. The six domains include physical health (4 items), psychological well-being (5 items), level of independence (4 items), social relation (4 items),environmental health (8 items), and spiritual health (4 items).Individual items were rated on a 5-point likert scale where 1 indicates low, negative perception and 5 indicate high, positive perceptions. In such a way, domain scores are scaled in a positive direction where higher scores denote higher QoL. However, seven facets are not scaled in a positive direction, meaning that for those facets, higher scores do not denote higher QoL. Those facets are re-coded in a positive direction so that high scores reflect better QoL. For instance, to what extent do you feel that physical pain prevents you from doing what you need to do? Options are 1-Not at all, 2-A little 3-A moderate amount 4-Very much and 5-An extreme amount. Facets like these were reversed into a positive direction so that 1 be an extreme amount in that order to 5-Not at all hence the higher scores will result into a good QoL. There are two questions of the WHOQoL-HIV BREF that examines the general QoL: question 12 asks about an individual’s overall perception of the QoL and question 13 asks about an individual’s overall perception of health. The first domain, physical health, deals with the presence of pain and discomfort, energy and fatigue, sleep and rest, and symptoms related to HIV. The psychological domain comprises negative and positive feelings, thinking, memory and concentration, body image and appearance, and self-esteem. Independent domain consists of mobility, activities of daily living, dependence on medication or treatments, and work capacity. The social relationships domain describes; personal relationships, social support, and sexual activity. Physical safety and security, home environment, financial resources, physical environment, and opportunities for acquiring new information were described under the environment domain. The last domain, spiritual health, contains information about concern about future death, forgiveness, and blame. There is also a general facet that measures the overall QoL of an individual (2 items asking individuals on the perception of their QoL and general health perception). The standard two weeks’ time frame was used to derive the patient's QoL experience.

As per the questionnaire authors’ suggestion, domain scores were calculated by computing the mean of the facet score within the respective domain and eventually multiplied by 4 to make domain scores comparable with the scores used in the WHO QoL-100 (WHOQOL-100), a commonly used scale [5, 33, 34].

Accordingly, the scores range from 4 to 20 points, reflecting the worst and the best QoL, respectively. The WHOQoL-HIV BREF user’s manual was rigorously followed for scoring and checking domain scores and score were transformed to the one that are found in the WHOQOL-100 then analyzed.

### Data analysis

Data entry, cleaning and analysis was done using SPSS statistical software version 23. Categorical variables were presented as frequencies and percentages. Mean and standard deviation was calculated for continuous variables. Prevalence of polypharmacy is reported as percentage. Study participants were divided into two groups; those with good QoL (WHO QoL-HIV BREF mean scores of > 50) and those with poor QoL (mean scores of ≤ 50) in overall QoL and general health perceptions facets.

Chi-square test was used to denote the association between independent variables (socio-demographic and clinical characteristics) and the outcomes of interest (polypharmacy and health related QoL (HRQoL). Univariate analysis using Poisson modified Poisson regression model was used to assess for the association between independent variables and the outcome of interest. Variables with *p* value of < 0.20 in the bivariate modified Poisson regression analysis were taken to the multivariate modified Poisson regression analysis. A *p*-value of < 0.05 in the multivariate model was considered statistically significant.

## Results

**Fig. 1:**
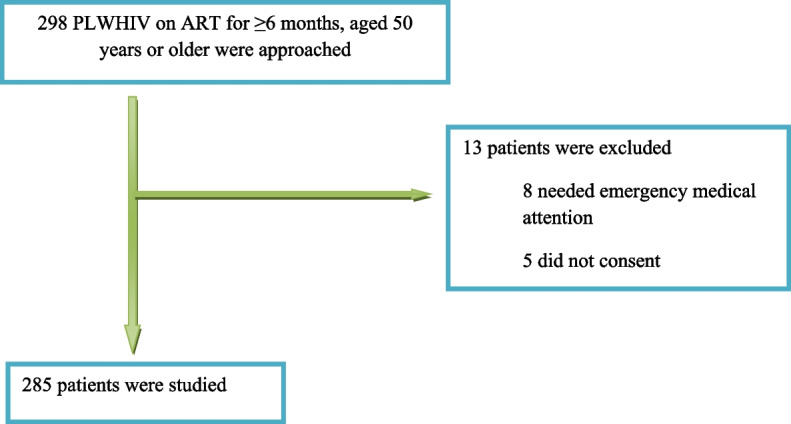
Flow chart showing enrollment of study participants

Figure 1 shows participants’ recruitment flow. A total of 298 PLWHIV aged 50 years or older and who were on ART for 6 months or more were screened for eligibility. Two hundred and eighty five participants fulfilled the inclusion criteria and were enrolled into the study.

### Socio-demographic and clinical characteristics

Table 1 shows the socio-demographic and clinical characteristics of 285 study participants. The mean age (± standard deviation) of study participants was 57(± 6.88) years. A total of 178 (62.5%) of the participants were female. Majority 266 (93.3%)of the study participants were residing in Dar es Salaam, were married 121 (42.5%) and had primary level education 142 (49.8%). Most 233 (81.8%) of the study participants were attending care and treatment clinics at Muhimbili national hospital, Upanga campus.

**Table 1:** Socio-demographic and clinical characteristics of the study patients, *N* = 285

Variable	Number n (%)
**Age groups in years**	
50–64	242 (84.9)
65 +	43 (15.1)
***Sex***	
Female	178 (62.5)
Male	107 (37.5)
**Residence**	
Dar es salaam	266 (93.3)
Other regions	19 (6.7)
**Marital status**	
Single	27 (9.5)
Married	121 (42.5)
Separated/ ivorced	81 (28.4)
Widowed	56 (19.6)
**Education**	
No formal/Primary	152 (53.3)
Secondary/College	133 (46.7)
**Health facility**	
MNH-Upanga	233 (81.8)
MNH-Mloganzila	52 (18.2)

*SD* Standard Deviation, *MNH* Muhimbili National Hospital, *HIV* Human Immunodeficiency Virus

### Prevalence and factors associated with polypharmacy

The prevalence of polypharmacyin this study population was found to be 18.2% (52/285).

Polypharmacy among older adults living with HIV on ART was found to be significantly associated with increasing number of co-morbidities, attendance to clinic(s) other than CTC and number of clinics attended other than CTC, all with a *p* value ≤ 0.001. There was no difference on the prevalence of polypharmacy between participants aged 50–64 years (16.9%) and those aged 65 years or older (26.6%), male (18.7%) and female (18.0%) participants, across the marital statuses or education level categories, all having *p* value > 0.05 (Table 2).

**Table 2:** Factors associated with polypharmacy among older adults living with HIV on ART at Muhimbili National Hospital in Dar es Salaam Tanzania *N* = 285

Variable	Polypharmacy			
	**Yes** Number (%)	**No** Number (%)	**Total** Number (%)	***p*-value**
**Age groups**				
50–64	41 (16.9)	201(83.1)	242 (84.9)	0.176
65 +	11(25.6)	32 (74.4)	43 (15.1)	
**Sex**				
Male	20 (18.7)	87 (81.3)	107 (37.5)	0.88
Female	32 (18.0)	146 (82.0)	178 (62.5)	
**Marital status**				
Single	4 (14.8)	23 (85.2)	27(9.5)	0.195
Married	27 (22.3)	94 (77.7)	121(42.5)	
Separated/Divorced	9 (11.1)	72 (88.9)	81(28.4)	
Widowed	12 (21.4)	44 (78.6)	56(19.6)	
**Residence**				
Dar es salaam	49 (18.4)	217 (81.6)	266(93.3)	0.774
Others	3 (15.8)	16 (84.2)	19 (6.7)	
**Education**				
No formal/Primary	24 (15.8)	128(84.2)	152 (53.3)	0.251
Secondary/post secondary	28 (21.1)	105(78.9)	133 (46.7)	
**Number of Co-morbidities**				
No co-morbidity	5 (2.7)	183(97.3)	188 (66.0)	< 0.001*
One	22(31.9)	47(68.1)	69 (24.2)	
Two or more	25(89.3)	3(10.7)	28 (9.8)	
**Clinics other than CTC**				
Yes	47(49.0)	49(51.0)	96 (33.7)	< 0.001
No	5(2.6)	184(97.3)	189 (66.3)	
**Number of clinics other than CTC**				
No other clinic	5(2.7)	183(97.3)	188 (66.0)	< 0.001*
One	22(31.9)	47(68.1)	69(24.2)	
Two or more	25(89.3)	3(10.7)	28(9.8)	

*NB*: *Denotes *p*-value for trend (p trend)

Predictors of polypharmacy were the number of co-morbidities, attendance of clinics other than the HIV clinic and thenumber of clinics participants had to attend. Compared to participants with no co-morbidities, the risk for polypharmacy was 18 times more among those with co-morbidities, PR (95% CI) = 18.09 (7.47–43.78), *p* < 0.001 (Table 3).

**Table 3:** Unadjusted and adjusted prevalence ratios and their corresponding 95% confidence intervals for the predictors of polypharmacy among the study population *N* = 285

**Characteristic**			**PR (95% CI)**	***P*-value**	**aPR(95% CI)**	***P*-value**
	Polypharmacy (%)	Total number				
**Age groups**						
50–64	11 (25.6)	242	1		1	
65 +	41 (16.9)	43	1.51 (0.84–2.70)	0.165	1.10 (0.69–1.75)	0.688
**Sex**						
Male	20 (18.7%)	107	1.04 (0.63–1.72)	0.88		
Female	32 (18.0%)	178	1			
**Residence**						
Dar	49 (18.4)	217	1.17 (0.40–3.40)	0.778		
Upcountry	3 (15.8)	16	1			
**Marital status**						
Without spouse	40 (17.5)	121	1		1	
With spouse	12 (21.4)	56	1.46 (0.90–2.39)	0.129	1.48 (0.99–2.21)	0.059
**Education level**						
No formal/ Primary	24 (15.8)	152	1			
Secondary/Post sec	28 (21.1)	133	1.33 (0.81–2.18)	0.254		
**Number of co-morbidities**						
No co-morbidity	5	188	1		1	
One or more	47 (48.5)	97	18.22 (7.48–44.38)	< 0.001	18.09(7.47–43.78)	< 0.001
**Clinic other than CTC**						
Yes	47 (49.0)	96	18.51 (7.59–45.07)	< 0.001		
No	5 (2.6)	189	1			
**Number of other clinics**						
Zero	7 (3.7)	190	1			
One or more	45 (47.4)	95	18.22 (7.48–44.38)	< 0.001		

*NB*: The variables clinics other than CTC and number of other clinics were not involved in multivariate analysis due to their co-linearity with number of co-morbidities. *PR* Prevalence ratio, *aPR* adjusted prevalence ratio

### Health related QoL and their predictors among older adult PLWHIV on ART

The overall QoL was good, WHOQoL HIV BREF mean ($\pm \mathrm{SD}$) score was 67.37 ± 11. Of the 285 participants, 40 (14%) reported poor QoL. Poor QoL was significantly more prevalent among participants with no formal or primary level of education (18.4%) compared to those with secondary or post-secondary education(9.0%), *p* = 0.023, among those with two or more co-morbidities (28.6%) compared to those with one (4.3%) and those without co-morbidities (15.4%), *p* < 0.01, and among participants who attended two or more clinics other than the CTC (27.6%) compared to those who attended the HIV clinic only (15.4%), *p* < 0.01 (Table 4).

**Table 4:** General quality of life for the study population by socio-demographic and clinical characteristics. *N* = 285

Variable	General QoL		Total	*P*-value
	Poor	Good		
**Age**				
50–64	35 (14.5)	207 (85.5)	242 (84.9)	0.622
65 +	5 (11.6)	38 (88.4)	43 (15.1)	
**Sex**				
Male	12 (11.2)	95 (88.8)	107 (37.5)	0.288
Female	28 (15.7)	150 (84.3)	178 (62.5)	
**Residence**				
Dar es salaam	35 (13.2)	231 (86.8)	266 (93.3)	0.111
Others	5 (26.3)	14 (73.7)	19 (6.7)	
**Marital status**				
Single	5 (18.5)	22 (81.5)	27 (9.5)	0.111
Married	13 (10.7)	108 (89.9)	121 (42.5)	
Separated/Divorced	9 (11.1)	72 (88.9)	81 (28.4)	
Widowed	13 (13.2)	43 (76.8)	56 (19.6)	
**Education**				
No formal/Primary	28 (18.4)	124(81.6)	152(53.3)	0.023
Secondary/Post-secondary	12(9.0)	121(91.0)	133(46.7)	
**Polypharmacy**				
Yes	10 (19.23)	42 (80.77)	52 (18.25)	0.233
No	30 (12.88)	103 (87.12)	233 (81.75)	
**Clinic other than CTC**				
Yes	11(11.5)	85 (88.5)	96 (33.7)	0.355
No	29 (15.3)	160 (84.7)	189 (66.3)	
**No. of co morbidities**				
None	29 (15.4)	159(84.6)	188(66.0)	< 0.01*
One	3(4.3)	66(95.7)	69(24.2)	
Two or more	8 (28.6)	20(71.4)	28(9.8)	
**No. of Clinics other than CTC**				
None	29(15.4)	159(84.6)	188(66.0)	< 0.01*
One	3(4.3)	66 (95.7)	69 (24.2)	
Two or more	8(27.6)	20 (72.4)	28 (9.8)	

^*^Denotes *p*-value for trend

Having no formal or primary education was the only factor that was associated with poor quality of life in univariate regression model for the study population. Multivariate regression analysis for predictors of QoL was not performed because only education qualified for that analysis (Table 5).

**Table 5:** Predictors of quality of life using modified poisson regression model

Variable	General QoL		Total	Univariate analysis RR (95% CI)	*P*-value
	**Poor (%)**	**Good (%)**			
**Age**					
50–64	35 (14.5)	207 (85.5)	242(84.9)	0.98 (0.93–1.04)	0.598
65 +	5 (11.6)	38 (88.4)	43 (15.1)	1	
**Sex**					
Male	12 (11.2)	95 (88.8)	107 (37.5)	1	0.270
Female	28 (15.7)	150 (84.3)	178 (62.5)	0.98 (0.98–1.02)	
**Residence**					
Dar es salaam	35 (13.2)	231 (86.8)	266 (93.3)	1	0.218
Upcountry	5 (26.3)	14 (73.7)	19 (6.7)	0.93 (0.83–1.04)	
**Marital status**					
Without spouse	27 (16.5)	137 (83.5)	164 (57.5)	1	0.271
With spouse	13 (10.7)	108 (89.3)	121 (42.5)	1.034 (0.97–1.09)	
**Education**					
No formal/Primary	28 (18.4)	124 (81.6)	152 (53.3)	0.896 (0.82–0.98)	0.021
Secondary/post sec	12 (9.0)	121 (91.0)	133 (46.7)	1	
**Polypharmacy**					
Yes	10 (19.23)	42 (80.77)	52 (18.25)	0.93 (0.80–1.07)	0.295
No	30 (12.88)	103(87.12)	233(81.75)	1	
**Clinic other than CTC**					
Yes	11 (11.5)	85 (88.5)	96 (33.7)	1.05 (0.95–1.14)	0.351
No	29 (15.5)	160 (84.5)	189 (66.3)	1	
**No of Co-morbidity**					
None	29 (15.4)	159 (84.6)	188 (66.0)	1	0.325
One or more	11 (11.3)	86 (88.7)	97 (34.0)	1.05 (0.95–1.15)	
**No of Clinic other than CTC**					
None	29 (15.4)	159 (84.6)	188 (66.0)	1	0.325
One or more	11 (11.3)	86 (88.7)	97 (34.0)	1.05 (0.95–1.15)	

## Discussion

In this study, the prevalence of polypharmacy among older adults PLWHIV on ART for 6 months or more was high at 18.2%. Polypharmacy was associated with increasing number of co-morbidities, attendance to clinic(s) other than CTC & number of clinics attended other than CTC. Polypharmacy was not associated with age, sex, marital status or education level. Only presence of co-morbidities could predict polypharmacy. Nearly ninety percent of the participants in the present study reported an overall good QoL. Having no formal education or primary education predicted poor QoL in the present study.

The prevalence of polypharmacy in this study was lower compared to a study done in Morogoro rural area in Tanzania which found the prevalence to be 24% among adults PLWHIV on ART [25]. The observed relatively lower prevalence in the present study could be due to differences in time periods the two studies were conducted, rural versus urban setting, socio-economic and the study population differences. One study in Uganda among HIV adults aged 50 years or older reported a prevalence of polypharmacy to be 15.3% [19] which is almost similar to the findings of our study. Other studies have reported different prevalence due to age, geographical, socio-economic factors and methodological difference with our study. For example a study done in Switzerland among Swiss HIV cohort aged 50 years or older found the prevalence of polypharmacy to be 31.8% [21].

Although not significant in the present study, polypharmacy was more prevalent among participants aged 65 years or older as compared to younger participants. Increasing age was found to be associated with polypharmacy in other studies [17, 35–37].

High prevalence of polypharmacy among older adults PLWHIV on ART in these studies are believed to be due to several possible reasons including differences in methodological approach [36, 38], racial differences and having health insurance [37]. The same observation has also been reported among HIV uninfected people aged 65 years or older [39, 40].

In the present study we found that having co-morbid conditions was independently associated with polypharmacy. This could be explained by the fact that, these co-morbidities which are both HIV related and non-HIV related do co-exist, with non-HIV related co-morbidities being on the rise due to increased life expectancy among PLWHIV [41–43]. Apart from individual factors that contribute to polypharmacy among this group there are also health system, facility-based and clinician factors including development on new therapies, increased preventive measures, medical guidelines and prescribing habits of the attending clinician [40, 44].

In the present study, the participants who reported poor QoL had the social domain of QoL most affected. The social domain can be affected by HIV-related stigma [45], not being sexually active and societal discrimination [46]. No formal and/or primary education level was found to be associated with poor QoL after controlling for other factors. Education level in the present study might have been confounded by other factors like unemployment and low income which weren’t checked in the present study.

Contrary to findings in other studies [5, 33], having co-morbidities was not associated with poor QoL in the present study. This could be because of differences in the study settings. For instance, a study by Zeleke et al.was done among admitted patients with HIV/AIDs in selected hospitals while the present study was done among relatively stable outpatients withHIV infection attending CTC [5]. In another study by Passos et al., the co-morbidities assessed were a bit different from those assessed in our study and this could be the main reason for disagreement between the studies. In the study by Passos et al., co-morbidities that were assessed included Hypertension, Diabetes, dyslipidemia, tuberculosis, hepatitis, CKD, COPD and cancer [33].

### Strength of the study

This study used a disease specific WHO tool to assess QoL among older adults PLWHIV on ART.

### Limitation of the study

As the study required participants to remember the number of medications in the past one week and QoL facets two weeks ago there was a possibility of recall bias.

## Conclusion

The prevalence of polypharmacy was high among older adults living with HIV. Polypharmacy was associated with increasing number of co-morbidities, attendance to clinic(s) other than CTC & number of clinics attended other than CTC. Having no formal education or primary education was found to be an independent determinant of poor QoL.

We recommend synchronization of treatment and care for HIV and HIV-unrelated co-morbidities in order minimize polypharmacy.

## Data Availability

The dataset is available from the corresponding author.
